# MicroRNA identification and expression analysis of wheat thermo-sensitive male sterile line BNS366 for fertility transformation

**DOI:** 10.3389/fpls.2025.1662041

**Published:** 2025-11-26

**Authors:** Junpeng Chen, Xue Hu, Ruilian Song, Genlou Sun, Sisi Huang, Wei Hua, Jinghuan Zhu, Daorong Zhang, Xifeng Ren

**Affiliations:** 1 Hubei Hongshan Laboratory, College of Plant Science and Technology, Huazhong Agricultural University, Wuhan, Hubei, China; 2 Xiangyang Academy of Agricultural Sciences, Xiangyang, Hubei, China; 3 Biology Department, Saint Mary’s University, Halifax, NS, Canada; 4 Agricultural Economics and Technology Research Institute, Hubei Academy of Agricultural Sciences, Wuhan, Hubei, China; 5 Zhejiang Academy of Agricultural Sciences, Hangzhou, Zhejiang, China

**Keywords:** wheat, miRNA, RNA-seq, male sterility, self-fruitfulness rate

## Abstract

**Introduction:**

Thermo-sensitive male sterile lines are the key element in the two-line hybrid system of wheat; some specific microRNAs (miRNAs) are involved in the development of anthers in plants, being an important cause of male sterility. Bai-Nong Sterility 366 (BNS366) is an excellent material for the study of thermo-sensitive male sterility, but no information is available on the role of miRNAs in regulating fertility in BNS366.

**Methods:**

In this study, miRNAs in the pollen mother cell periods and the tetrad periods of the low-temperature sterile and normal fertile anthers of BNS366 were characterized using RNA sequencing (RNA-seq).

**Results:**

MiRNA sequencing identified 22 differentially expressed known miRNAs and eight novel miRNAs with the largest differential expression folds. The prediction of target genes of the 30 miRNAs yielded 25 target genes, which were highly expressed in wheat spike, and mainly regulated by 13 miRNAs. Gene Ontology (GO) analysis showed that these target genes mainly related to DNA replication and transcription. Five miRNAs (miR9662a, miR5062, miR9662b, miR9653a, and miR9672b) showed opposite expression patterns with their respective potential target genes in BNS366 anther of different fertility. *TraesCS5D02G192700* is a potential target gene for miR5062 and encodes an argonaute protein, which functions in meiotic prophase germ cell development and maintenance of meiosis.

**Conclusion:**

Our findings suggest that the miR5062-argonaute module may plays a pivotal role in the fertility transformation of BNS366. Based on these results, the miRNA-target gene regulatory network involved in the fertility restoration of BNS366 at low temperatures was proposed.

## 1 Introduction

Wheat (*Triticum aestivum* L.) is one of the most important cereal crops in the world, being a vital source of food for the world’s population (Hassan et al., 2021). Male sterility in plants with both male and female reproductive organs is described as a condition where the pistil develops normally but the stamen fails to produce pollen due to a developmental abnormality (Xu et al., 2023; Chen et al., 2024). Currently, male sterile lines have been widely used in many crops, and their yield and quality have significantly improved, such as rice (*Oryza sativa* L.), oilseed rape (*Brassica napus* L.), maize (*Zea mays* L.), and soybean [*Glycine max* (L.) Merr.] (Marchant and Walbot, 2022). Although many male sterile lines have been found in wheat, it has not yet been extensively employed in production; consequently, there is a need to elucidate the mechanisms underlying the failure of male fertility in wheat, with the aim of enhancing wheat production and quality and promoting the efficient development of wheat cultivation (Appels et al., 2018; Melonek et al., 2021; Song and Hedgcoth, 1994; Brownfield, 2021). Bai-Nong Sterility (BNS) is a thermo-sensitive male sterile line of wheat, and BNS366 was selected by saturation backcrossing between BNS and Zhengmai 366 (Chinese certified wheat variety), with the same pattern of fertility transition as BNS (Liu et al., 2021). Previous studies have proved that BNS366 has the characteristics of thermo-sensitive male sterility, its sterility trait is stable, and the sensitive period of its fertility transition is from the differentiation of floret primordia to the formation of the connective septum (Zhou et al., 2014, 2015).

MicroRNAs (miRNAs) are endogenous non-coding small RNA (sRNA) molecules, approximately 18–25 nt in length, that function as key regulators in eukaryotes and mediate post-transcriptional gene silencing by base pairing with complementary sequences on target mRNAs, leading to translational repression or mRNA degradation (Xu et al., 2020). Numerous studies have indicated that miRNAs play a crucial role in regulating male fertility and fertility restoration under high-temperature (HT) or low-temperature (LT) stress in various plants. In barley (*Hordeum vulgare* L.) and *Arabidopsis thaliana*, studies have demonstrated that HT stress leads to a specific decrease in endogenous auxin levels within developing anthers, resulting in anther abortion; some heat-responsive miRNAs, including miR160, miR167, and miR393, have been reported to be involved in the auxin signal transduction pathway and may be involved in this process of male sterility (Sakata et al., 2010; Sun, 2012). In soybean, Ding et al. (2023) demonstrated that the miR156b–*GmSPL2b* module may mediate reactive oxygen species (ROS) clearance by regulating flavonoid metabolism, thereby restoring fertility in cytoplasmic male sterile lines under HT stress. In rice, research has revealed that miR2118 and miR2275 can bind to *OsAGO1d* in pollen mother cells to mediate phasiRNA synthesis, thereby maintaining fertility under LT stress (Si et al., 2023). In cotton (*Gossypium hirsutum* L.), mechanistic models from previous studies suggest that HT stress suppresses the expression of miR156 and miR8643, leading to the upregulation of two membrane receptor-like protein kinase genes, *PERK12* and *RBK1*, which compromises pollen fertility stability; meanwhile, HT-induced miR167 and miR390 may disturb oxygen homeostasis by inhibiting the expression of related genes in a ROS scavenging system, ultimately resulting in anther indehiscence and male sterility (Ding et al., 2017; Zhang et al., 2023).

High-throughput sequencing is a common and effective method for identifying miRNAs that affect male sterility in plants. Jiang et al. (2021) identified 15 differentially expressed miRNAs and predicted that miR159 and its target genes may be involved in the regulatory network of pollen development and male sterility. Fang et al. (2014) identified a total of 156 known miRNAs and 24 novel miRNAs in two ponkan mandarin (*Citrus reticulata* Blanco) lines. Zhang et al. (2021) analyzed miRNAs and their targets in soybean cytoplasmic male sterility and its maintainer line, and a regulatory network was proposed based on male sterility differentially expressed genes. There have been similar studies in wheat; for example, Bai et al. (2017) used RNA sequencing (RNA-seq) to examine the expression profiles of miRNA in the spikelets of male sterility wheat line Beijing Sterile 366 (BS366), a total of 100 known miRNAs exhibited significant differential expression between three different developmental stages, and their target genes were predicted and finally verified using degradation analysis. Sun et al. (2018) used small RNA sequencing and degradome analysis to identify miRNAs and their targets in the male sterile wheat line 337S. A total of 102 unique miRNAs were identified, and it was predicted that tae-miR1127a, with its target gene *SMARCA3L3*, may play an important role in the male sterility process. Han et al. (2021) performed miRNA-seq on the male sterile wheat line YS3038 and identified 140 differentially expressed miRNAs in three stages of wheat development. They predicted 79 differentially expressed target genes and found the *TaeRPK* gene to be possibly involved in the fertility conversion of YS3038.

In recent years, BNS366 has become an excellent material for researchers to study male sterility, but most of the studies have focused on the macroscopic level, for example, the effect of plant hormones on fertility and pollen sterility (Liu et al., 2024, 2018; He et al., 2014); the role of miRNAs in BNS366 fertility transformation is not addressed. In this study, we characterized the differential expression of miRNA from the anthers of wheat line BNS366 under low-temperature sterile and normal fertile conditions using RNA-seq. We screened out miRNAs with significant differential expression during another development and their target genes. We identified five candidate genes affecting the fertility transition of BNS366 and five miRNAs regulating these target genes, thus providing useful information for further studying the sterility mechanism of the thermo-sensitive wheat sterile line BNS366.

## 2 Materials and methods

### 2.1 Plant materials and growth conditions

The experimental material used in this study was the thermo-sensitive male sterile wheat line BNS366. For RNA sequencing, plants were planted on two sowing dates: 20 October 2020 and 20 January 2021. Furthermore, to explore its temperature-dependent sterility, plants were sown at four different time points: 10 October 2021 (I), 12 November 2021 (II), 15 December 2021 (III), and 15 January 2022 (IV).

All materials were planted in the experimental field of Huazhong Agricultural University, Wuhan, Hubei Province, China (N30°32′ and E114°20′). Planting was conducted in 10-row zones at a time with a 10-m line length and a 20-cm row distance. Irrigation and fertilization followed normal field management practices for wheat production.

### 2.2 Sampling period and self-fruitfulness rate

**Figure 1:**
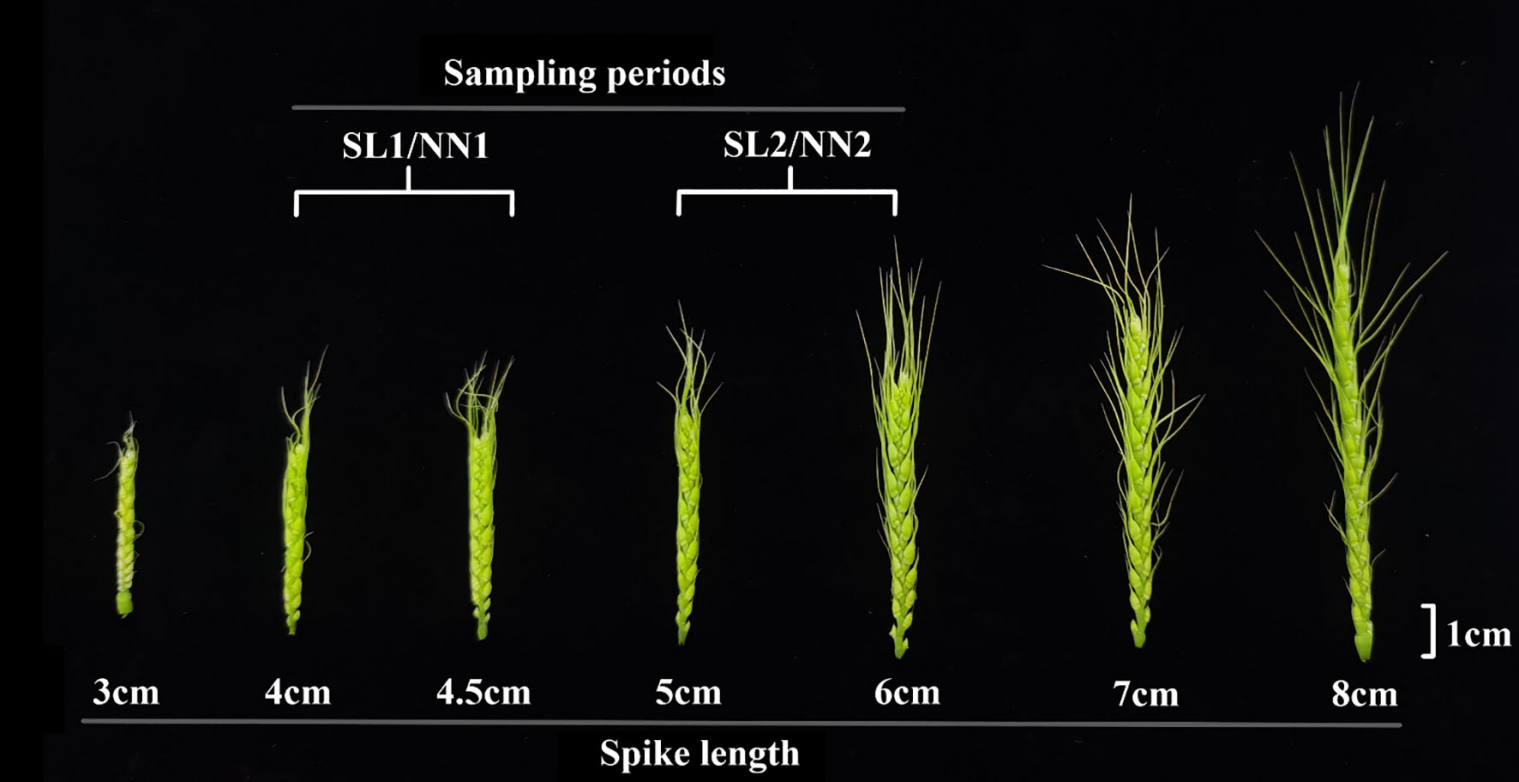
The spike phenotype from NN and SL plants of BNS366 at different developmental stages. Bar = 1 cm. NN, normal day-length/normal temperature; SL, short day-length/low temperature.

The determination of the wheat stamen development period was based on spike length according to Sun et al. (2018). Previous studies have demonstrated that pollen mother cells in BNS366 begin to exhibit abnormalities during the mid-metaphase I (He et al., 2014). In this study, young wheat spikes measuring 4–4.5 and 5–5.5 cm (corresponding to the pollen mother cell periods and the tetrad periods, respectively) were selected from sowing dates 20 October 2020 and 20 January 2021. From each group, 15 spikes were harvested, and the anthers were isolated for RNA-seq analysis (**Figure 1**). The sowing dates of 20 October 2020 and 20 January 2021 were categorized as representing short day-length/low temperature (SL) and normal day-length/normal temperature (NN) environments, respectively. Therefore, these four samples were named SL1 and NN1 (pollen mother cell period) and SL2 and NN2 (tetrad period).

At the monocarpic leaning stage in wheat (wheat awn protrudes ≤1 cm from the flag leaf sheath), 10 spikes were taken from each spike at 10 a.m. for fixation, pollen grains were stained and prepared using iodine-potassium iodide (I_2_-KI) solution, and iodine-stained wheat BNS366 pollen from different periods was analyzed for pollen fertility using microscopic observation.

The sterility of wheat BNS366 was determined using an index of self-fruitfulness (Murai, 2001). At each sowing date (I–IV), nine BNS366 plants were selected, and all spikes from each plant were bagged before anthesis to prevent random pollination. Upon maturity, the bagged spikes were harvested. The self-fruitfulness rate for an individual plant was calculated as the average across all its spikes, and the overall self-fruitfulness rate for each sowing period was then determined by averaging the rates from all nine plants. Self-fruitfulness rate = (number of grains per spike/number of effective florets per spike) × 100%.

### 2.3 Construction of small RNA libraries

Total RNA was extracted from anthers using the FastPure Universal Plant Total RNA Isolation Kit (Vazyme, Nanjing, China). The samples included sterile and fertile anthers collected at both the pollen mother cell stages and the tetrad stages, with three biological replicates per group, constituting a total of 12 samples. RNA integrity and purity were assessed using the Agilent 2100 Bioanalyzer and a spectrophotometer. High-quality RNA samples (OD260/280 = 1.8~2.2, OD260/230 ≥ 2.0, RNA integrity number (RIN) ≥ 8, 28S:18S ≥ 1.0) were employed for sequencing library construction.

Libraries were constructed using the Vazyme miRNA 1st Strand cDNA Synthesis Kit (Vazyme, China). The procedure was performed following the manufacturer’s instructions. Briefly, the procedure involved adding a poly(A) tail to the 3′ end of miRNAs, followed by first-strand cDNA synthesis using a universal reverse transcription primer. Four sequencing libraries of cDNA were sent to the Shanghai Mindray Biotechnology Co., Ltd., Shanghai, China for sequencing on an Illumina, San Diego, USA HiSeq 2500 platform. A total of 12 samples were sequenced, each with a sequencing depth of 10-Mb raw reads.

### 2.4 Raw read preprocessing, genome matching, and quality control

Raw sequencing data were processed to obtain clean reads by removing low-quality sequences, 5′ adapter contaminants, repetitive sequences, and reads with overly simple base composition. Subsequently, the clean reads were aligned to the reference genome using HISAT2 (v2.0.4) with default parameters. Finally, to enable cross-comparison of gene expression levels both within and between samples, read counts were normalized to transcripts per million (TPM). Principal component analysis (PCA) was performed using the prcomp function in the stats package that comes with the R language, and graphing was performed using the ggplot function in the ggplot2 package.

### 2.5 Identification of miRNAs and screening of differentially expressed miRNAs

The miRBase22.0 database (http://www.mirbase.org/) was used as a reference to find known miRNAs. Then, small RNAs were compared with the Rfam database and Repbase database to filter ribosomal RNAs (rRNAs), transfer RNAs (tRNAs), small nuclear RNAs (snRNAs), small nucleolar RNAs (snoRNAs), other ncRNAs, and repetitive sequences. The miRDeep2 and RNA Folding Form V2.3 (https://www.unafold.org/mfold/applications/rna-folding-form-v2.php) were used to predict small RNAs with Dicer cleavage sites. The non-annotated ones in the previous steps were labelled as novel miRNAs. The expression level of each miRNA was calculated according to the TPM read method. The expression levels of miRNAs were normalized by TPMs using the formula: normalized expression = mapped reads/total reads × 1,000,000 (Zhou et al., 2010). Finally, differentially expressed genes were analyzed using DESeq2, and the fold-change (FC) was calculated based on the TPM value. The screening of differentially expressed miRNAs was performed by setting a threshold of |log2FC| > 0.585 and *p*-value ≤ 0.05 (DEseq2/edgeR/Limma). Differentially expressed genes were visualized and analyzed using the Hiplot online research data visualization tool (https://hiplot.cn).

### 2.6 qRT-PCR analysis

The expression levels of miRNAs and genes were detected using qRT-PCR. Specific primers were designed using oligo software, tested for specificity using the NCBI website (https://www.ncbi.nlm.nih.gov), and synthesized by Wuhan Tianyi Huiyuan Biotechnology Co., Ltd, Wuhan, Hubei, China. The primers used for qRT-PCR analysis are shown in **Supplementary Table S1**. qRT-PCR was performed using the SYBR Premix EX Taq™ II (Perfect Real-Time) kit (Takara, Shiga, Japan), and the operating steps were as follows: the PCR system contained 1 μL cDNA, 5 μL 2× miRNA qPCR Mix (with SYBR Green), 0.2 μL forward primer (10 μM), 0.2 μL reverse primer (10 μM), and 3.6 μL ddH_2_O. The cycling parameters were 94°C for 30 s, 40 cycles of 94°C for 5 s, and 60°C for 30 s. *TaActin* was used as an internal control, and the 2^−ΔΔCt^ method was used to determine the relative transcript abundance. The upstream primers used for miRNA amplification in qRT-PCR analysis were miRNA-specific primers, designed in accordance with the instructions provided in the company’s miRNA reverse transcription kit. The downstream primers were the universal primers provided in this kit.

### 2.7 MiRNA target gene prediction

The target genes of the miRNAs were predicted using the psRobot (Wu et al., 2012). The wheat cDNA file on the Ensembl plants web (https://plants.ensembl.org) was downloaded, IWGSC RefSeq v1.1 was used as the reference genome, default parameters were used, the target penalty score was 2.5 (lower is better), and the maximal number of permitted gaps was 1. The transcript data of these target genes were downloaded from the Wheat Multiomics Centre (http://wheatomics.sdau.edu.cn) to analyze their expression levels in each organ.

## 3 Results

### 3.1 Sowing periods’ effect on BNS366 stamens, pollen, and spikes

**Figure 2:**
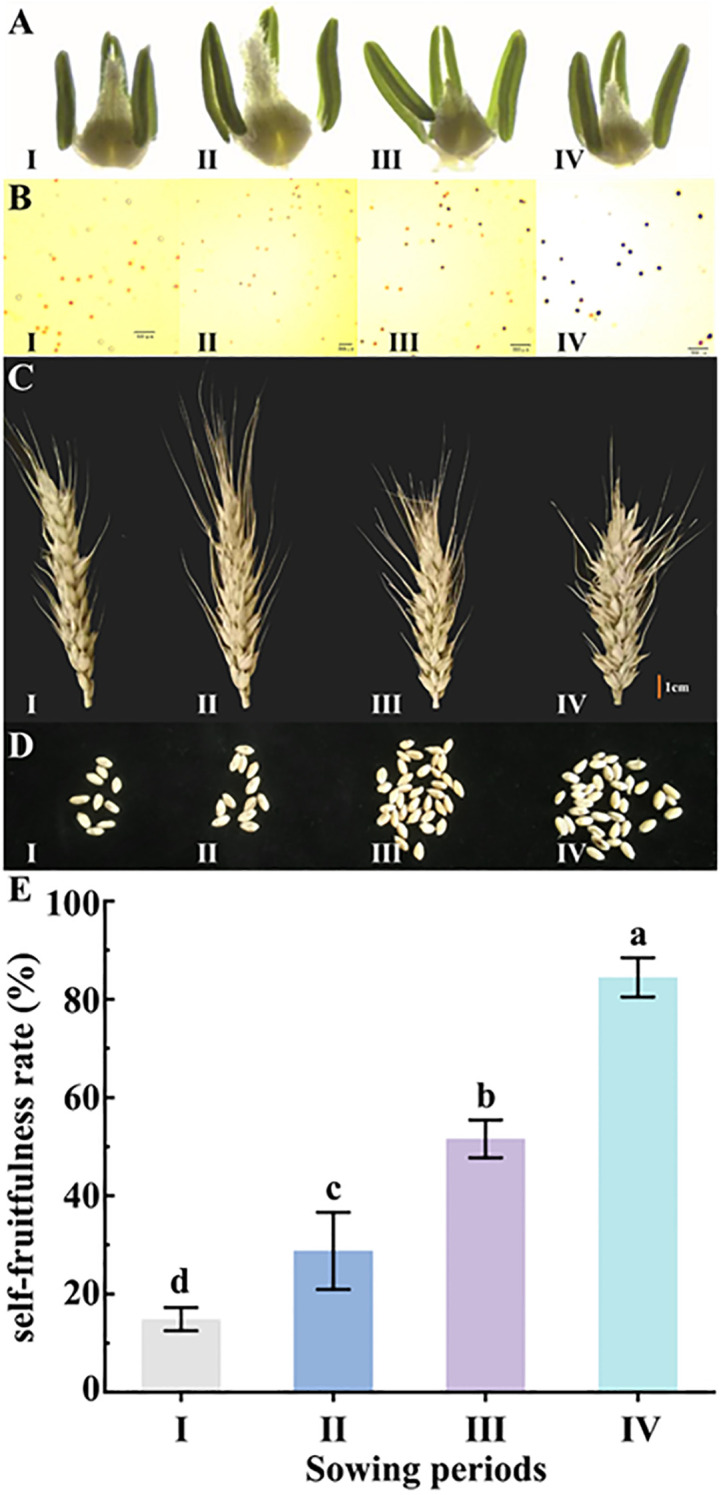
Observations on phenotypes and the self-fruitfulness rate of wheat BNS366 at different sowing periods. **(A)** Stamen development in the mononuclear stage of BNS366. **(B)** BNS366 stained with pollen iodine solution I2-KI at flowering stage. **(C)** Morphological characteristics of the spike at maturity of BNS366. **(D)** Number of grains per spike at mature stage for BNS366. **(E)** Self-fruitfulness rate of BNS366 under different sowing periods. I, first sowing period; II, second sowing period; III, third sowing period; IV, fourth sowing period.

The wheat sterile line BNS366 showed different agronomic phenotypes at different sowing periods. Compared to periods II–IV, period I displayed pronouncedly shorter and more wilted stamens (**Figure 2A**). Pollen staining indicated that period I showed complete abortion with wilted pollen, and pollen from period II was highly abortive; pollen from period III was also half abortive, but pollen from period IV was hardly abortive (**Figure 2B**). In the observation of BNS366 spikes at different sowing periods, it was found that spikes from periods I and II were longer and thinner and set fewer grains compared to those from periods III and IV (**Figures 2C, D**).

### 3.2 Sowing periods’ effect on BNS366 self-fruitfulness rate

Statistical analysis revealed a significant effect of sowing period on the self-fruitfulness rate of BNS366. The self-fruitfulness rate for period I was only 14.87%, but this increased to 28.77% for period II, 51.58% for period III, and 84.47% for period IV, which was nearly equivalent to the normal fertility range. The self-fruitfulness rate among the four sowing periods had a highly significant difference (**Figure 2E**). For BNS366, the environmental variable for the sowing period main response is temperature, so it can be deduced that the self-fruitfulness rate of BNS366 increases with increasing temperature, which is, in general, consistent with the phenotype of BNS366 pollen and spikes changing with temperature. All these results indicate that BNS366 exhibits male sterility at low temperatures.

### 3.3 Overview of the small RNA sequencing

**Figure 3:**
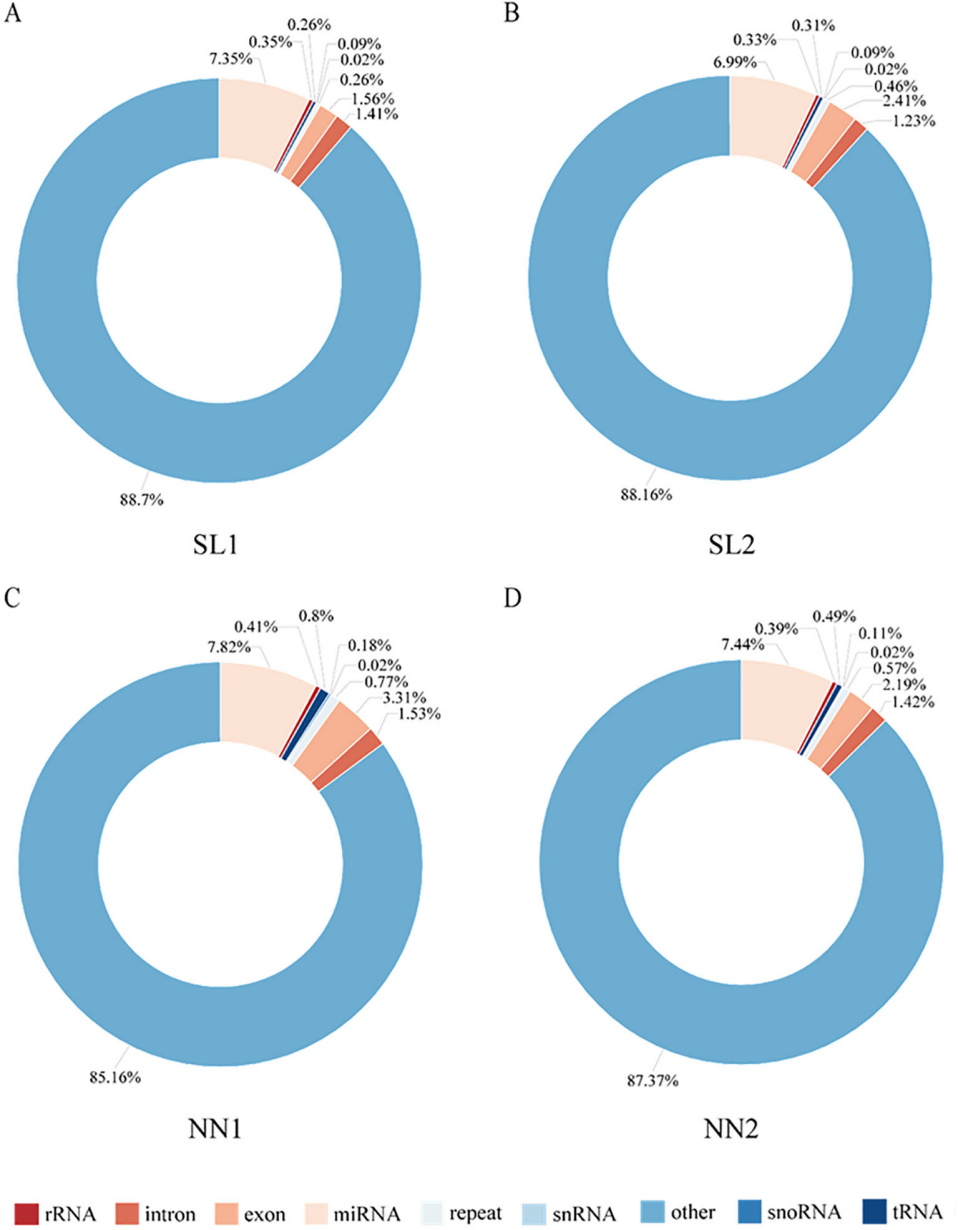
Annotation and classification of sRNA in four libraries. **(A)** sRNA annotation in the SL1 library. **(B)** sRNA annotation in the SL2 library. **(C)** sRNA annotation in the NN1 library. **(D)** sRNA annotation in the NN2 library. NN, normal day-length/normal temperature; SL, short day-length/low temperature.

To identify differentially expressed miRNAs, 12 small RNA libraries from pollen mother cells and tetrads at both fertile and sterile stages were constructed. Following the assembly and annotation of these libraries, a total of 1.63 billion original reads were obtained, with an average of 0.54 billion original reads. After filtering the data, 161 million effective reads were obtained (**Supplementary Table S2**). Comparison of the Rfam and Repbase databases revealed the presence of five types of non-coding RNA: miRNA, rRNA, tRNA, snoRNA, and snRNA. Notably, the proportional distribution of each non-coding RNA type was highly consistent across the different libraries. For example, miRNA accounts for the highest proportion in the four libraries, ranging from 6.99% to 7.82%, while snRNA accounts for the lowest proportion at 0.02% in all four libraries (**Figure 3**).

**Figure 4:**
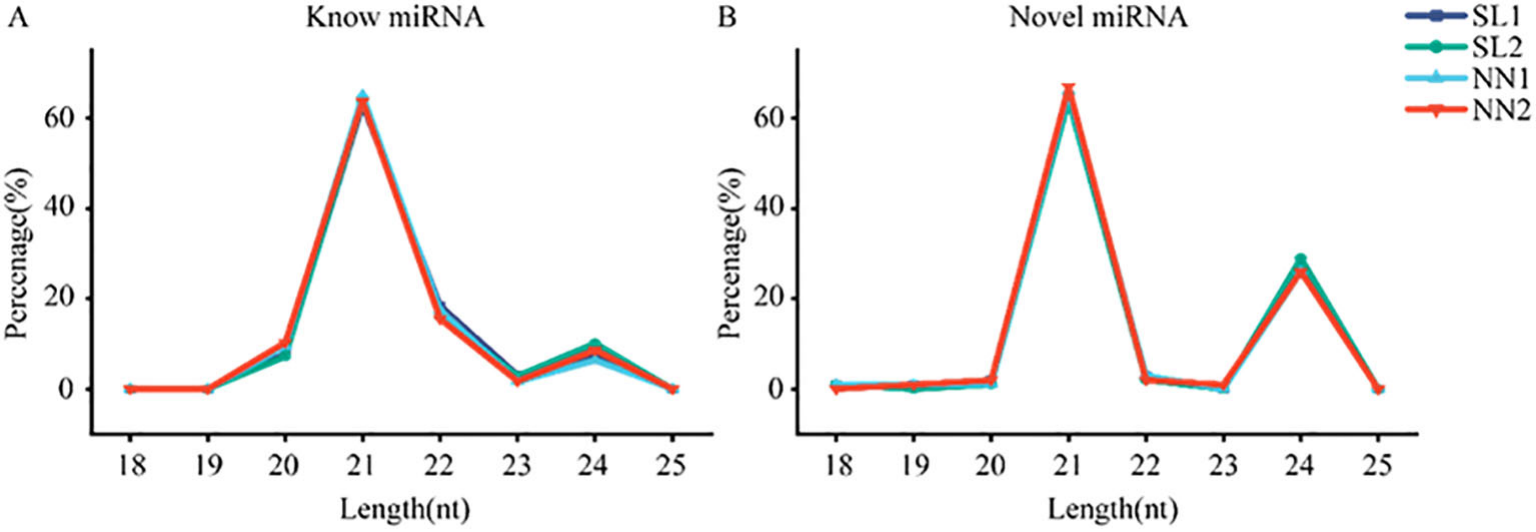
Length distribution of miRNA. **(A)** The length distribution of known miRNA in SL1, SL2, NN1, and NN2. **(B)** The length distribution of novel miRNA in SL1, SL2, NN1, and NN2. NN, normal day-length/normal temperature; SL, short day-length/low temperature.

**Figure 5:**
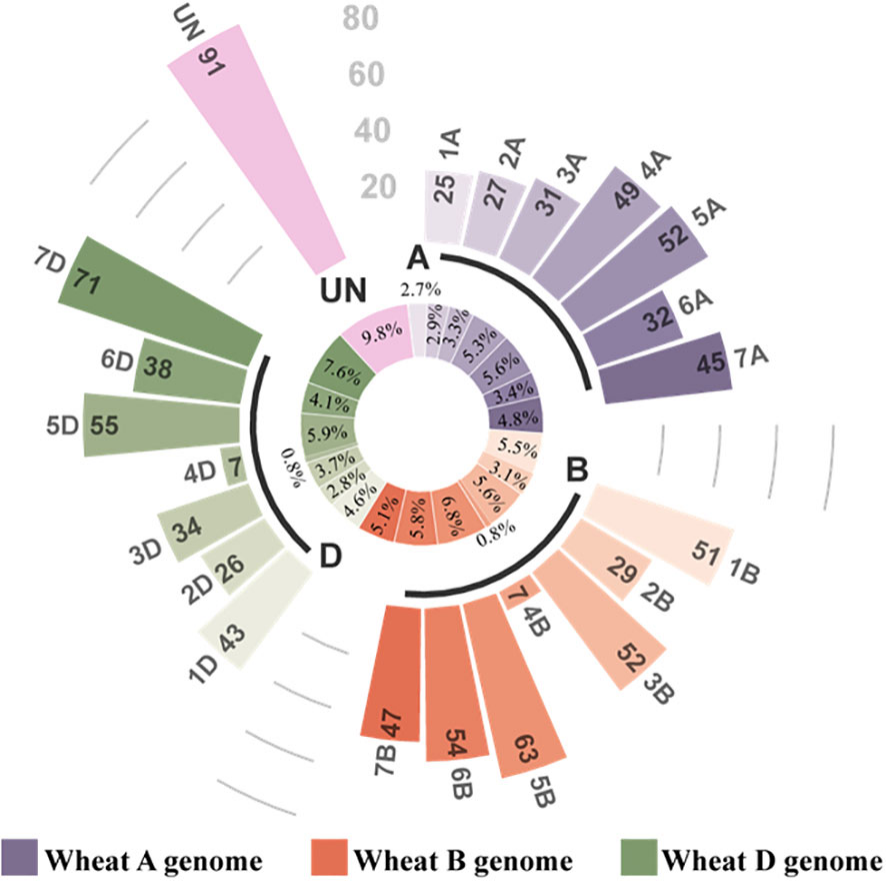
Distribution of miRNAs on chromosomes. The inner circle is a circular diagram representing the ratio of miRNAs to total miRNAs on each chromosome. The outer circle is a histogram representing the number of miRNAs on each chromosome.

The miRNAs obtained from the libraries were analyzed. The miRNAs were matched to the plant miRbase21.0 database, and the wheat genome sequence called known miRNAs; only those that matched the wheat genome sequence were considered novel miRNAs. A total of 929 miRNAs were identified in the four libraries using sRNA sequencing, comprising 81 known miRNAs (**Supplementary Table S3**) and 848 novel miRNAs (**Supplementary Table S4**, **Table 1**). The lengths of the miRNAs obtained by RNA sequencing were primarily distributed within the range of 20–24 nt, with the highest percentage of 21-nt miRNAs (**Figure 4**). Of 929 identified miRNAs, 848 unknown function novel miRNAs could not be classified; 47 of 81 known miRNAs were classified into 29 different miRNA families (**Supplementary Table S5**). Finally, the distribution of these miRNAs on wheat chromosomes was analyzed (**Figure 5**); 929 miRNAs are distributed across all chromosomes in wheat, with 261, 303, and 274 miRNAs in the A, B, and D chromosome groups, respectively. However, the distribution across the A, B, and D chromosome groups was uneven, with the 7D chromosome having the most miRNAs (71) and the 4B and 4D chromosomes having the fewest (7).

**Table 1:** Statistical analysis of all miRNAs.

MiRNA type	Known miRNA	Novel miRNA	Total miRNA
miRNA of SL1	65	842	907
miRNA of SL2	64	828	892
miRNA of NN1	69	843	912
miRNA of NN2	58	821	879

**Figure 6:**
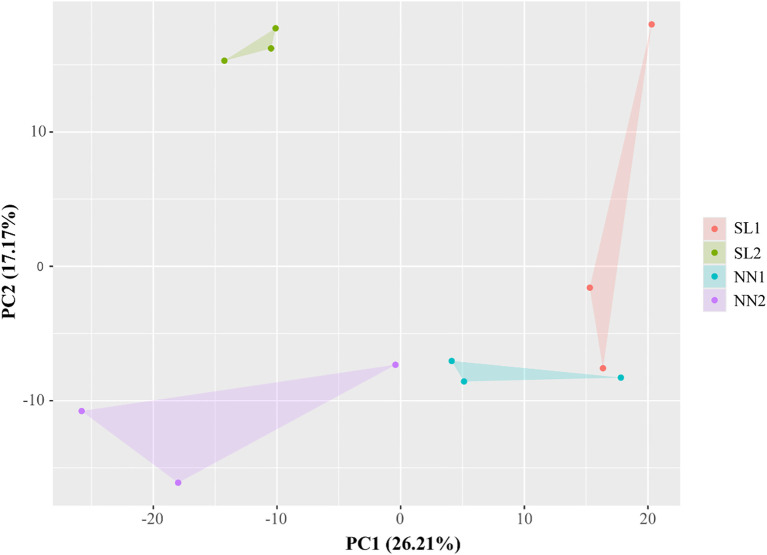
PCA of 12 RNA-seq libraries. The figures within the brackets on the horizontal and vertical axes denote the principal component contribution rates for the first and second principal components, respectively. PCA, principal component analysis.

PCA was conducted on the 12 miRNA libraries to evaluate inter-group distinctions and intra-group reproducibility. The distance between points in the figure represents the degree of similarity between samples. PC1 refers to the top contribution rate, which is the factor that has the greatest influence on variation, and PC2 is the second factor. **Figure 6** shows that the four different samples are fully distinguishable at the 2D level. SL2 and NN1 have better inter-group reproducibility, but SL1 and NN2 have poor inter-group reproducibility. Overall, sample duplication within the group was consistent, and the PCA data showed the high quality of biological repetition of the samples.

### 3.4 Identification of novel miRNAs

To validate the predicted novel miRNAs, six novel miRNAs, including 1D_6425, 2A_6922, 2B_8724, 4B_21959, 6D_35192, and 7A_39175, were selected randomly for confirmation using qRT-PCR. The results indicated that all selected miRNAs were expressed at varying levels across different periods and conditions (**Supplementary Figure S1A**). In addition, six novel miRNAs with perfect secondary structures and Minimum Free Energy (MFE) were predicted using RNA Folding Form V2.3; the results showed that the hairpins of these novel miRNA precursors did not contain large internal loops or bulges, and their precursors’ MFE mainly concentrated in −16.30 to −32.70 kcal mol^−1^ (**Supplementary Figure S1B**).

### 3.5 Identification of differentially expressed miRNAs in wheat

**Figure 7:**
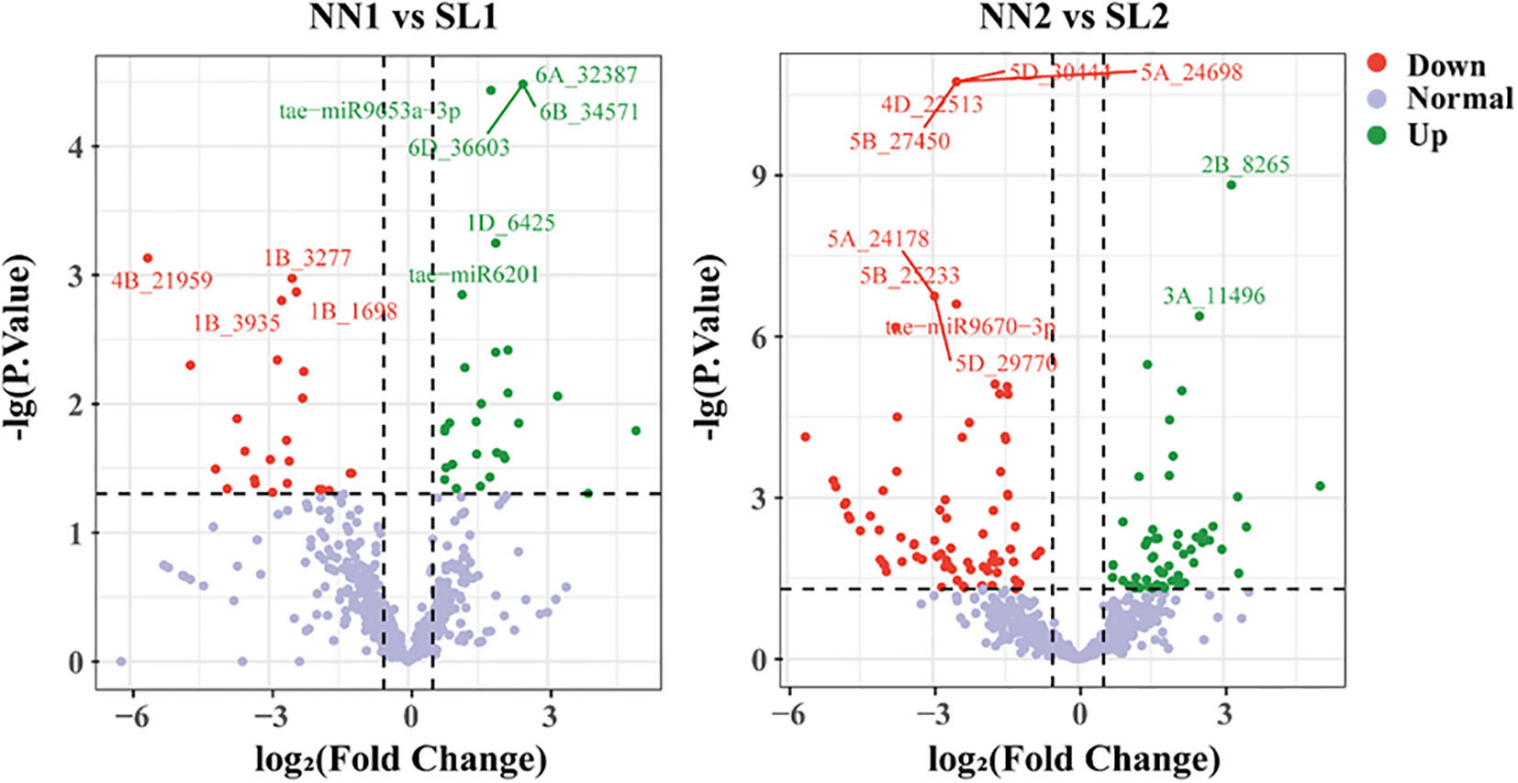
Differentially expressed miRNAs.

In order to identify the differential expression of miRNAs associated with the BNS366 infertility mechanism, the expression profiles of miRNAs were analyzed in four libraries using the criteria of |log_2_FC| > 0.585 and *p*-value ≤ 0.05 (**Figure 7**). Twenty-two known miRNAs and eight novel miRNAs with the highest expression levels were identified (**Supplementary Table S6**, **Supplementary Table S7**). The majority of miRNAs have been previously reported to be associated with fertility and to exhibit differential expression patterns between the fertile and sterile growth stages, suggesting that these miRNAs may play a role in regulating the fertility of wheat line BNS366. To evaluate the reliability of the sequencing results, the expression levels of 12 miRNAs associated with fertility were validated using quantitative real-time polymerase chain reaction (qPCR); the results aligned closely with the results of the small RNA sequencing, indicating that the data generated from the sRNA sequencing were reliable (**Supplementary Figure S2**).

### 3.6 Targets of differentially expressed miRNAs and functional analysis

**Figure 8:**
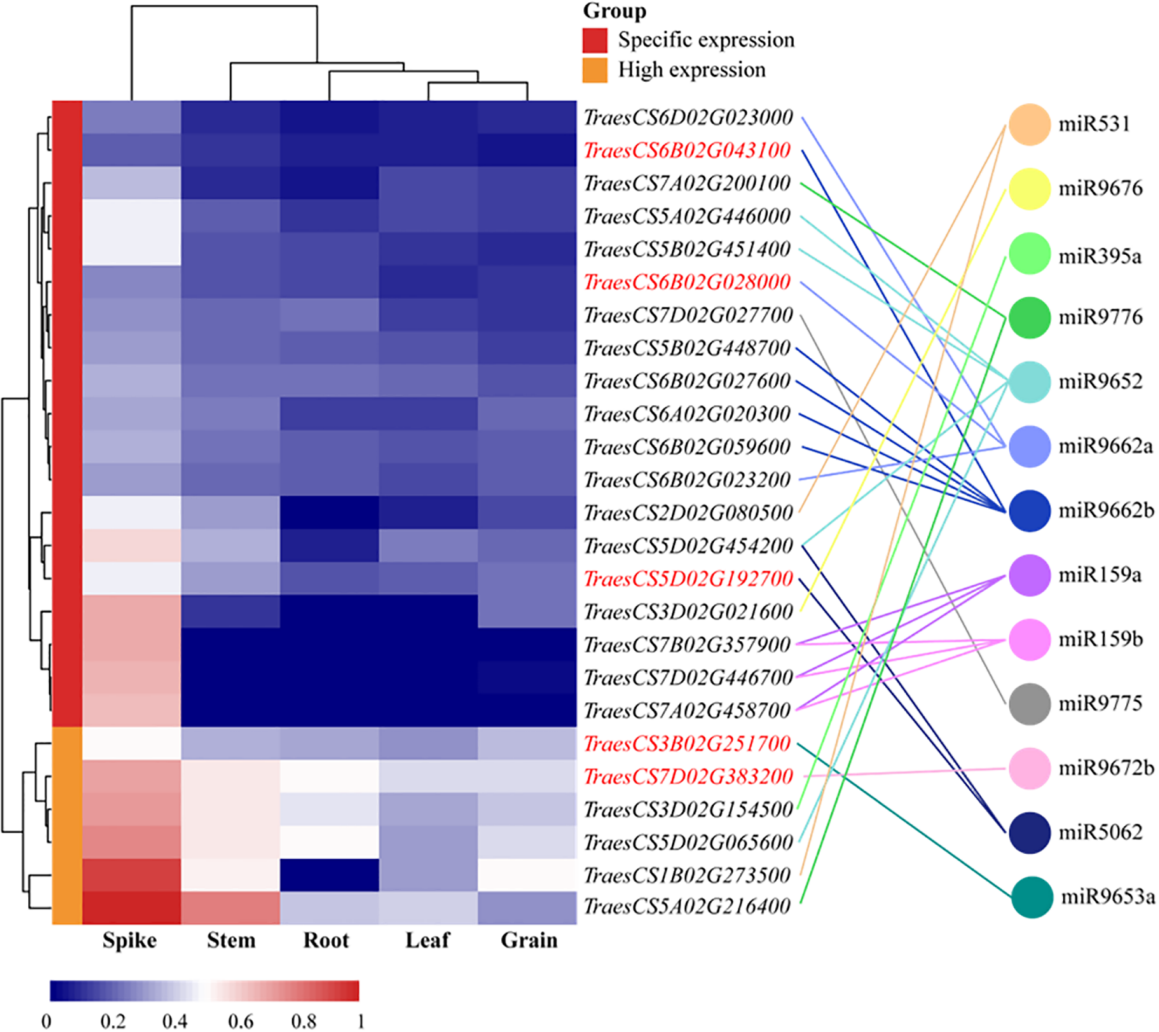
Analysis of target gene expression patterns in different tissues.

**Figure 9:**
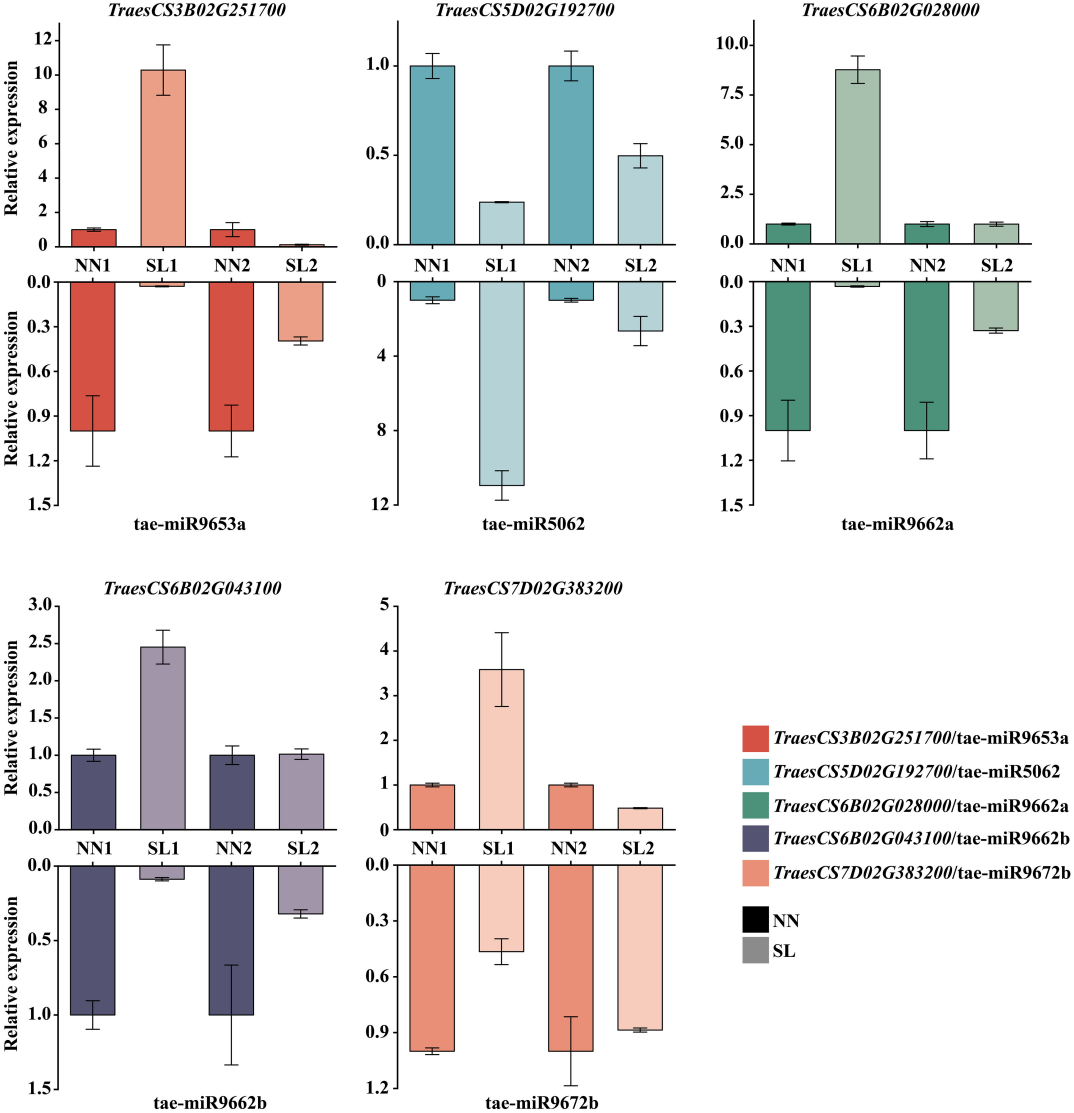
Expression level of miRNAs and target genes by qPCR. Note: The upper columns represent the target gene expression level in SL1, SL2, NN1, and NN2. The lower columns represent the miRNA expression level in SL1, SL2, NN1, and NN2. NN, normal day-length/normal temperature; SL, short day-length/low temperature.

To characterize the functions of differentially expressed miRNAs, their target mRNAs were predicted using the psRobot software. A total of 1,086 target transcripts were obtained as potential targets of 30 candidate differentially expressed miRNAs (DEMs) (**Supplementary Table S8**). Subsequently, to ascertain whether these target genes were involved in the regulation of miRNA activity in wheat, the transcription data for these target genes were downloaded from the wheat Multi-omics Center, and the expression levels of the genes corresponding to 1,086 target transcripts were analyzed in the roots, stems, leaves, spikes, and grains, which led to the identification of 25 target genes that exhibited high or specific expression in the spike, which were found to be predominantly regulated by 13 miRNAs (**Figure 8**). GO functional enrichment analysis was performed on the 25 target genes obtained using the DAVID database (https://davidbioinformatics.nih.gov/); a total of two molecular functions, three biological processes, and three cellular components were obtained; and the predicted target genes were enriched in the DNA-templated transcription termination, chloroplast organization, regulation of DNA-templated transcription, DNA binding, and double-stranded DNA binding (**Supplementary Table S9**). Based on the GO analysis results, 15 genes potentially involved in DNA replication were further identified. These genes were regulated by seven distinct miRNAs, with miR9662b controlling the largest number at five genes, and miR9652 and miR9662a each regulate three genes, while the remaining miRNAs govern only one gene each (**Supplementary Table S10**). To further elucidate the regulatory relationship between miRNAs and target genes, qPCR was employed to assess the expression levels of 15 target genes in the spike of the BN366 cultivar under different developmental conditions. By analyzing the co-expression patterns of miRNAs and their corresponding target genes (**Supplementary Table S10**), a total of five target genes (*TraesCS6B02G02800*, *TraesCS5D02G192700*, *TraesCS6B02G043100*, *TraesCS3B02G251700*, and *TraesCS7D02G383200*) were ultimately selected, which are potentially regulated by miR9662a, miR5062, miR9662b, miR9653a, and miR9672b, respectively; for example, miR5062 is upregulated in both the SL1 and SL2 libraries, but its target gene *TraesCS5D02G192700* is downregulated in both of these libraries. MiRNA and its target gene expression level in the library are opposite, and it is considered that the target gene may be regulated by the miRNA (**Figure 9**). The structures of the five miRNAs and their alignments with their target transcripts are shown in **Supplementary Figure S3**.

## 4 Discussion

### 4.1 Conserved and non-conserved miRNAs affecting reproductive development in wheat

Previous studies have shown that conserved miRNAs such as miR156, miR159, miR160, miR164, miR166/165, miR167, miR169, miR172, and miR319 play critical roles in plant flower development (Luo et al., 2013). In this study, miR159a, miR159b, miR160, and miR319 were also identified. The non-conserved tae-miR9652-5p had been identified as differentially expressed in other studies of male sterility and was predicted to decrease wheat fertility, possibly by repressing the expression of meiosis arrested at *leptotene 1* (*MEL1*) genes (Sun et al., 2018). In this study, in addition to tae-miR9652-5p, tae-miR9652-3p was identified; both belong to the same family and were found to be differentially expressed in the tetrad stage, but no valuable target genes were identified. The three miRNAs tae-miR9653a-3p, tae-miR396-5p, and tae-miR9677b were identified in this study and also by Li et al. (2019). Among them, the non-conserved miRNA tae-miR9653a-3p, differentially expressed in the uninucleate stage, the conserved tae-miR396-5p, and the non-conserved tae-miR9677b were suggested to potentially affect different target genes at the uninucleate stage to influence fertility (Li et al., 2019). Other miRNAs identified in this study, such as conserved miR395, were mostly mined in sulfate-deficient stress studies, and few studies have pointed to their effect on plant fertility (Yang et al., 2022; Mu et al., 2018). Our results, alongside those of previous studies, suggest the existence of a complex regulatory network in plant reproductive development. It may be the case that disruption to the gene expression homeostasis of this regulatory network will result in irreparable damage to wheat fertility (Sun et al., 2018).

### 4.2 Some of the novel miRNAs identified were consistent with previous studies

To further enhance the credibility of the novel miRNAs identified in this study, we compared them with those previously identified in wheat male sterility research, and we found that 39 mature sequences in our study corresponded to 33 of Han et al. (2021), only one corresponded to the identified eight novel miRNAs of Sun et al. (2018), and only four mature sequences from this study matched 689 novel miRNAs reported by Li et al. (2019). The lower number of aligned novel miRNAs may have resulted from differences in the analysis software used, variations in experimental materials, or potentially higher false-positive rates in software-predicted novel miRNAs (**Supplementary Table S11**).

### 4.3 Tae-miR5062 may be involved in maintaining normal meiosis by regulating TaMEL1

In this study, miR5062 target *TraesCS5D02G192700* encodes an argonaute protein, which is a homologue of the rice *MEL1* (Zhan, 2021), and was named *TaMEL1*. In rice, the *MEL1* has been demonstrated to be involved in premeiotic germ cell development and meiosis processes to maintain the meiotic progression. It is highly expressed exclusively during the pollen mother cell phase and meiosis prophase, with gene expression being repressed once meiosis is complete (Jiang et al., 2020; Nonomura et al., 2007; Zhang et al., 2020). This study has the same conclusion as previous research (Lian et al., 2021). Therefore, we hypothesized that the abnormal increase in miR5062 expression during the pollen mother cell period under low-temperature sterility conditions in BNS366 suppressed the expression of its target gene *TaMEL1*, which resulted in a deficiency of argonaute protein to maintain the normal progression of meiosis in pollen mother cells, and ultimately led to the abortion of BNS366. In addition, we suspect that there may be a class of upstream regulatory genes of miR5062 involved in this regulatory network. In the future, we will reveal the regulatory relationship between miR5062 and TaMEL1 and refine this regulatory network.

### 4.4 Replication factors C1 and AGAMOUS protein may also be involved in the abortion process of BNS366

The tae-miR9672b target gene *TraesCS7D02G383200* was highly homologous to rice *replication factor C* (*OsRFC1*) and *Arabidopsis AtRFC1*; these two genes play a role in DNA double-strand break repair during meiosis in rice and *Arabidopsis* cells (Xia et al., 2007; Qian et al., 2018). The target gene of tae-miR9653a, *TraesCS3B02G251700*, has a CCCH zinc-finger construct domain and is highly homologous to the *Arabidopsis* gene *AtHUA1*. It has been shown that *AtHUA1* is involved in flower development and facilitates the processing of mRNA precursors by binding to the AGAMOUS (AG) protein (Cheng et al., 2003). Therefore, we suggested that the tae-miR9653a–*TraesCS3B02G251700* interaction may regulate the process of flower development, thus affecting the fertility of BNS366.

**Figure 10:**
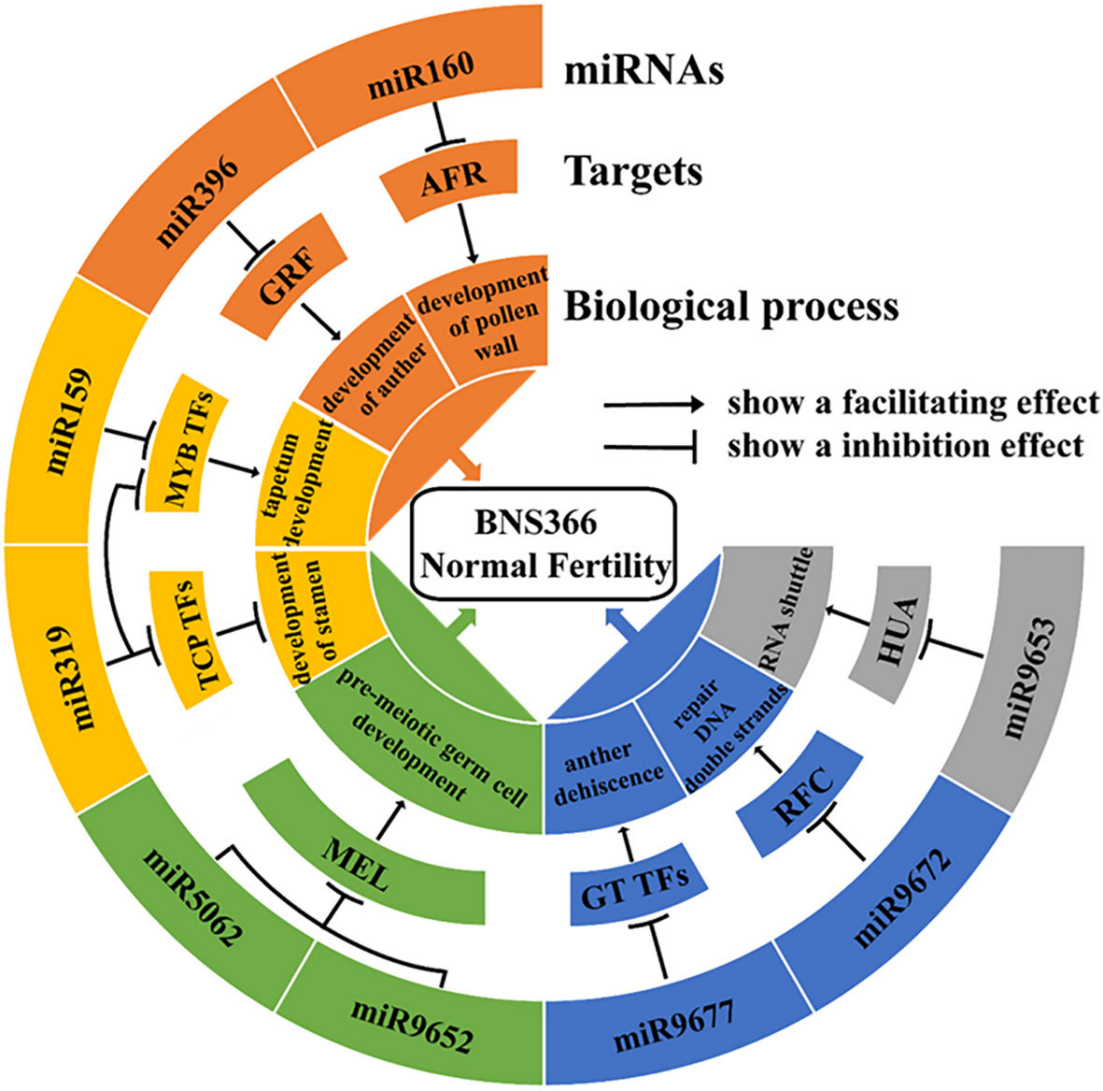
Pathway map of miRNAs regulating BNS366 fertility.

It was reported that miR160 could regulate a group of *ARF* genes: *ARF10*, *ARF16*, and *ARF17*. In particular, *ARF17* exerts the most significant influence on the reproductive development of plants (Hao et al., 2022). Wang et al. (2017) showed that *ARF17* expression under miR160 regulation is critical for its function during another development in *Arabidopsis*; the overexpression of *ARF17* led to defects in microsporocytes and tapetum. Previous studies have demonstrated that the miR396-GRF pathway is critical for the early morphogenesis of stamen and pollen mother cells (Baucher et al., 2013). The miR159 targets include *MYB* transcription factors; among these, *GAMYB-like* genes have been demonstrated to be involved in flower and anther development in rice, *Arabidopsis*, barley, and rye (Millar and Gubler, 2005; Luo et al., 2013). Murray et al. (2003) reported that the overexpression of *HvGAMYB* led to male sterility in barley; previous studies have demonstrated that miR319 simultaneously targets *TCP4*, *MYB33*, and *MYB65* to influence *Arabidopsis* stamen development, with *TCP4* being the primary target gene of miR319. The overexpression of *TCP4* results in severe developmental defects in stamens (Nag et al., 2009). Although *MYB33* and *MYB65* are miR319 target genes, they remain primarily regulated by miR159, with miR319 exerting only a relatively minor regulatory effect upon them (Palatnik et al., 2007). Based on the experimental results obtained in this study and previously published results, we constructed a miRNA–target gene schematic to explain the miRNA regulatory network involved in the restoration of fertility of BNS366 at high temperatures (**Figure 10**).

### 4.5 Significance and limitations of this study

Sterile lines have always been one of the most important topics in crop breeding (Longin et al., 2012). Using thermo-sensitive male sterile lines is the key to two-line hybridization in wheat. Therefore, analyzing the molecular mechanism of thermo-sensitive male sterile lines is conducive to the development of two-line hybrid breeding, which has great significance to the production of wheat (Niu et al., 2023).

Although this study has significant findings, it still has shortcomings; for example, sampling at different periods may have made environmental temperature a variable, potentially leading to the identification of certain differential miRNAs due to temperature differences rather than affecting male sterility. Another limitation is the lack of further investigations into the novel miRNAs.

## 5 Conclusions

In conclusion, we used high-throughput sequencing technology to characterize the expression profiles of miRNAs in wheat thermo-sensitive sterile line material BNS366 during the fertility conversion process. Using qPCR analysis revealed that five miRNAs (miR9662a, miR5062, miR9662b, miR9653a, and miR9672b) and their potential regulatory target genes showed completely opposite expression patterns in different fertile anthers, which indicated that they may participate during male fertility transition. In particular, the argonaute protein-encoding gene *TraesCS5D02G192700* maintained high expression levels during early spike, suggesting that it may play important roles in the fertility transformation of BNS366.

## Data Availability

The RNA sequencing data generated in this study have been deposited in the NCBI Sequence Read Archive (SRA) database under accession number PRJNA1358924.
