# Supplementary material for: MicroRNA identification and expression analysis of wheat thermo-sensitive male sterile line BNS366 for fertility transformation
Source: Front Plant Sci. 2025 Nov 26;16:1662041. doi: 10.3389/fpls.2025.1662041 (PMC12689989; doi:10.3389/fpls.2025.1662041)
Supplement: Supplementary file 1 [file DataSheet1.zip › Supplementary Materials/Figure S1.Relative expression, perfect secondary structures and MFE of six novel miRNAs.docx]

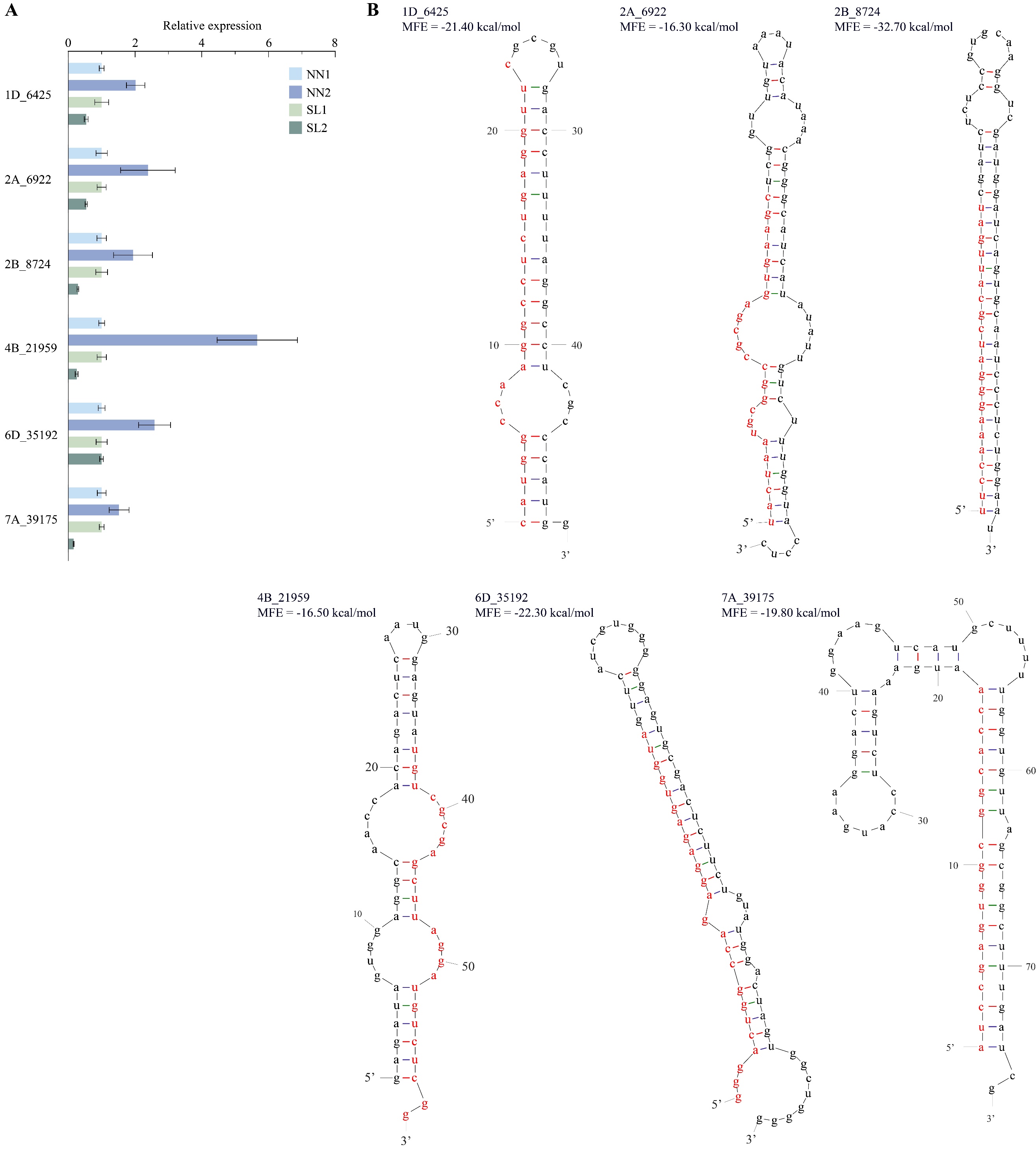


**Figure S1.** Relative expression, perfect secondary structures and MFE of six novel miRNAs. (a) The relative expression of six novel miRNAs. SL1: Pollen mother cell period under sowing period I; SL2: Tetrad period under sowing period I; NN1: Pollen mother cell period under sowing period IV; NN2: Tetrad period under sowing period IV. (b) The perfect secondary structures and MFE of six novel miRNAs. Red letters denote the mature sequence of this miRNA.
