# Supplementary material for: MicroRNA identification and expression analysis of wheat thermo-sensitive male sterile line BNS366 for fertility transformation
Source: Front Plant Sci. 2025 Nov 26;16:1662041. doi: 10.3389/fpls.2025.1662041 (PMC12689989; doi:10.3389/fpls.2025.1662041)
Supplement: Supplementary file 1 [file DataSheet1.zip › Supplementary Materials/Figure S2. miRNAs expression level between qPCR and RNA-seq.docx]

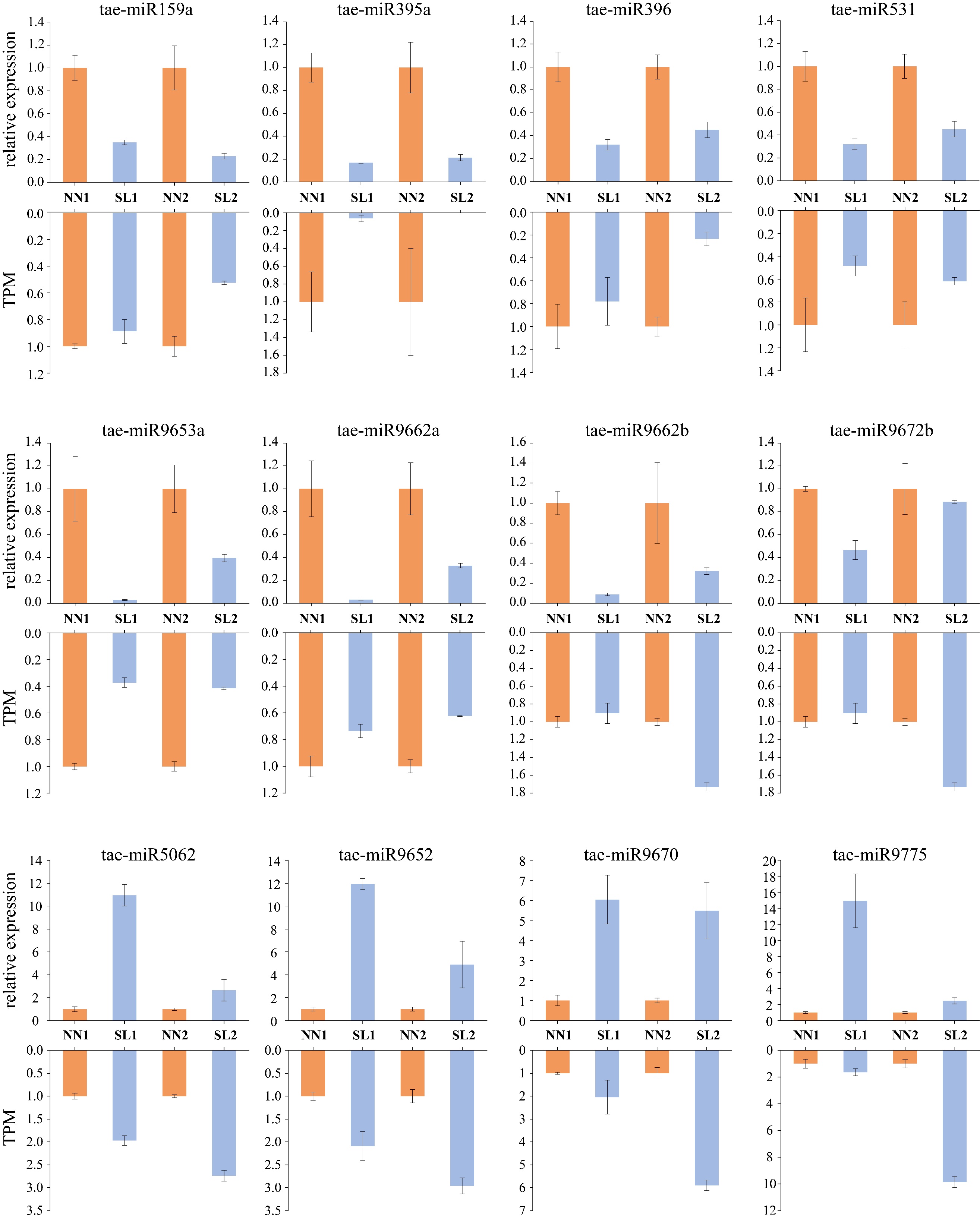


**Figure S2.** miRNAs expression level between qPCR and RNA-seq. The blue column represented the miRNA expression level in NN1 and NN2, the orange column represented the miRNA expression level in SL1 and SL2. Above the quantitative results of miRNA qPCR, and below were the results of miRNA sequencing.
