# Supplementary material for: MicroRNA identification and expression analysis of wheat thermo-sensitive male sterile line BNS366 for fertility transformation
Source: Front Plant Sci. 2025 Nov 26;16:1662041. doi: 10.3389/fpls.2025.1662041 (PMC12689989; doi:10.3389/fpls.2025.1662041)
Supplement: Supplementary file 1 [file DataSheet1.zip › Supplementary Materials/Figure S3. Schematic representation of the binding of five miRNAs with opposite expression patterns to their anchored target gene mRNAs.docx]

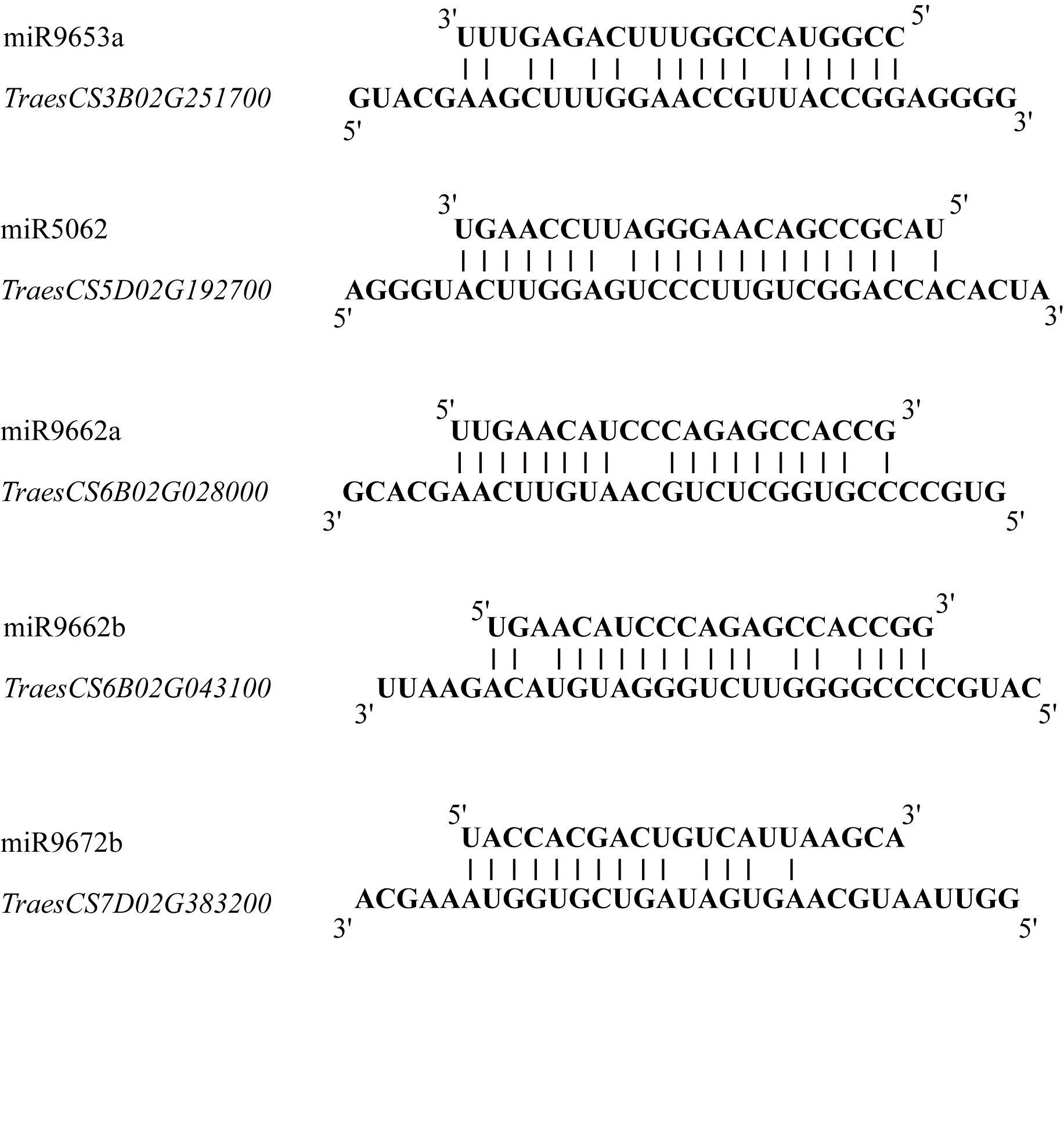


**Figure S3.** Schematic representation of the binding of five miRNAs with opposite expression patterns to their anchored target gene mRNAs.
